# Shame and development of self: a relational, cognitive, and linguistic perspective

**DOI:** 10.3389/fpsyg.2025.1683750

**Published:** 2026-01-12

**Authors:** Filippo Saccardo, Alessandro Grecucci, Irene Messina, Valerie Lesk, Laura Franchin

**Affiliations:** 1 Department of Psychology and Cognitive Science, University of Trento, Trento, Italy; 2 Universitas Mercatorum, Rome, Italy; 3 Faculty of Management Law and Social Sciences, Department of Psychology, University of Bradford, Bradford, United Kingdom

**Keywords:** attachment, developmental psychology, language acquisition, narrative, self-conscious emotions, shame, sociomoral development, storytelling

## Abstract

Shame is a pivotal component in the development of sociomoral cognition, emerging at the intersection of self-awareness, social evaluation and internalized norms. Its emergence corresponds with significant developmental milestones, including the formation of attachment, the acquisition of language, and the development of Theory of Mind. Collectively, these factors provide a framework for the evolution of the sense of self. As children begin to represent themselves through the perspectives of others, shame functions as a regulatory mechanism, reinforcing social coherence and moral alignment. This review integrates findings from developmental psychology, cognitive neuroscience and cultural theory in order to elucidate the dual structure of shame: self-referential yet socially mediated. From a multi-faceted perspective, the article presents shame not simply as an emotion but as a dynamic process through which individuals navigate, monitor, and shape their moral identity within relational and cultural contexts.

## 1 Introduction

O shame, where is thy blush?

—Shakespeare, *Hamlet*, 3.4.81

In Greek mythology shame makes its appearance, together with justice, with the precise purpose of restoring social harmony and enables humans to live in communities. Protagoras, in the homonymous platonic dialogue, tells that Prometheus stole practical wisdom and fire from the gods to give it to humankind. However, he did not bring them the political technique with the consequence that man was able to survive but did not know the art of living together. Everywhere reigned conflicts. Zeus, fearing for the extinction of mankind, sent Hermes who dispensed justice and shame (*dìke* and *aidōs*) to each individual; anyone who did not conform to social norms would be killed (Plato, 1997, ca. 388 B.C.E./1997, 321d-322d). The concept of shame is the prominent aspect of the Greek term *aidōs* which is a word with great semantic richness^1^ (e.g., Cairns, 1993). It also indicates the need to adhere to social norms, the need to remain within shared social limits and norms: who do not respect the boundaries of honor (*timé*) experience *aidōs*. If we consider the Homeric epic, for example, we can see how heroes feared “losing face” and arousing public indignation (Homer, 1984, ca. VIII-VI c. B.C.E/1984, VI 340-346, XXII 103-106; Hooker, 1987). The specific role of *aidōs* led Dodds (1951) to argue that the Homeric world expressed a culture of shame (which would later be transformed into a culture of guilt in 5th-century BC under the influence of a series of factors that cannot be explored in detail here; e.g., Cairns, 1993; Dodds, 1951). Since antiquity, the theme of shame focused on the individual and their place within social relationships; the Greek hero who fails to follow heroic ethics is indeed subject to the community's gaze. The essence of shame lies in its dual nature; it refers both to the self and to the social context in which the self develops. Any attempt to study it from a psychological perspective must therefore include both of these elements.

While it remains challenging to provide a universally accepted definition of emotion (Lakoff, 2016; Mulligan and Scherer, 2012; Scherer, 2005) and there is theoretical disagreement on which emotions are considered basic (Ekman, 1992) or primary (Barrett, 2006; Ortony and Turner, 1990), there is a broad consensus in defining shame as a self-conscious emotion. Self-conscious emotions arise from self-evaluations and reflect how individuals perceive their actions, thoughts, and behaviors in relation to social and moral standards (e.g., Lewis, 2008; Tangney, 2012). These emotions share a dual nature in that they are intrinsically self-referential, focusing on one's sense of self, and are deeply intertwined with the social context, as they are shaped by external norms and interpersonal interactions (Robins and Schriber, 2009; Sznycer, 2019).

We examine the developmental implications of this dual nature of shame. As a self-conscious emotion, shame arises during the same stage of development in which children are building their self, acquiring language and social skills, and developing the ability to understand others' thoughts and emotions (Mascolo and Fischer, 1995; Nikolić et al., 2023). This includes the mentalizing development and the capacity to make inferences about others (Asgarizadeh et al., 2023; Schwarzer et al., 2024). By the time these milestones have been fully achieved, children are capable of experiencing shame in a more sophisticated and evolved form, reflecting their growing cognitive, social, and emotional capacities (Erikson, 1950; Harter, 2012; Tangney and Dearing, 2002). The simultaneous development of a sense of self, mentalization, and the experience of shame suggests a closely interdependent relationship between these constructs (Asgarizadeh et al., 2023; Fonagy et al., 2018). Building on this perspective, this review explores the developmental trajectories of shame in connection with the evolving sense of self, which is understood to emerge through interpersonal interactions within a social context. Our goal is to offer a broad, integrative perspective on the literature, bridging insights from various branches of cognitive science to create a comprehensive understanding of these related phenomena.

In this review, we use a tree structure that begins with a broad overview of self-conscious emotions, focusing specifically on shame and its interpretive models. From this broad perspective, we move toward a deeper exploration of the roots of shame within the development of a sense of self. We focus specifically on: (a) the emergence of a sense of self through early interpersonal interactions, leading to the construction of internal working models; (b) cognitive self in terms of ideal self, associated with Theory of Mind development; and (c) language development and narrated self.

## 2 Shame

### 2.1 Self-conscious emotions

Self-conscious emotions require self-awareness (Duval and Wicklund, 1972) and the capacity for self-representation. According to the distinction outlined by Tracy and Robins (2004), basic emotions provide adaptive advantages for survival and are consequently expressed universally (Ekman and Friesen, 1971, 1976), showing distinct physiological markers such as quick activation, difficulty of suppression, and specialized expressive and hormonal patterns. Self-conscious emotions, however, are primarily involved in constructing one's sense of identity and in motivating and regulating cognition and behavior in social environments. Moreover, unlike basic emotions (Ekman, 1992; Ekman and Friesen, 1971, 1976), the expression of self-conscious emotions is not discrete or universally recognizable; they can manifest differently across individuals and cultures (Shweder, 2003). The interpretation and encoding of these emotions are primarily behavioral, encompassing the individual's overall posture and body language (Lewis, 2008). Furthermore, they are cognitively complex: they require representational capacities of mentalization and internalization of others' points of view. With regard to their evolutionary development, while primary emotions emerge in the first 9 months of life (happiness, surprise, fear, sadness, anger, disgust; Ekman, 1992), with precursors already in the neonatal stage (the pleasure/joy system, the fear system, the anger system; Sroufe, 1995), self-conscious emotions require years for full maturation (e.g., Tracy et al., 2007). They typically occur around the age of 3 years and fully mature in the school period (Lewis, 2008). Mascolo and Fischer (1995) observed specific behaviors that correlated with shame in children around the age of two and a half to three namely, gaze avoidance, closed posture (head down slumped, collapsed body, arched shoulders, hands under the table, arms or hands in front of face or body). Feeling shame drives the child to avoidance, to concealment, whereas guilt implies a desire to repair the harm, a prosocial behavior (Barrett et al., 1993). Moreover, according to Lewis (2008), shame requires an important element to be experienced: the presence of a spectator who is perceived as judging one's behavior. Therefore, the mechanism of shame involves elaborate cognitive skills such as metacognition.

### 2.2 Internal and external shame

In 2003, Gilbert identified two types of shame – internal and external. The first arises as a result of negative self-evaluations and focuses the attention on the self with (threat) judgements and feelings about oneself. The latter is the result of an evaluation or a criticism made by a third party. To explain further, external shame focuses on the mind of the other with a desire to repair reputation, and avoid or hide the self. This aspect is closest to the idea of shame provided by Goffman (1959), according to whom, this emotion would emerge from the perception of having failed to meet others' expectations or having violated social norms. Therefore, this aspect can function as a kind of moral compass, guiding the individual toward a change in which the self strives to converge with a socially ideal image of itself.

According to Gilbert (2003), the key difference between external shame and guilt lies in their focus and emotional experience. External shame arises from the perception that others view one negatively. It is the feeling that one is perceived as flawed, inadequate or unworthy by others, leading to feelings of rejection and humiliation, and a desire to hide or withdraw. The focus is on how one is perceived by others and the fear of social exclusion or negative judgement. External shame concerns the social self and the threat to one's social standing or acceptance within a group. In the field of linguistic pragmatics, Tantucci (2021) uses the term “extended intersubjectivity” to describe a speaker's orientation toward a third party or generalized social audience. This concept involves paying attention to the broader social mind, including the norms, expectations, beliefs, and evaluative stances attributed to a community or a non-present observer. Guilt, in contrast, is an emotion centered on a specific behavior or action that has caused harm to another person. It involves recognizing that one has done something wrong and feeling bad about it, which can prompt reparative or prosocial actions (e.g., Drummond et al., 2017). Rather than being rooted in a threat to one's social worth, guilt is rooted in the moral evaluation of one's actions and their impact on others.

Misailidi (2020) showed the connection between self-attributed self-conscious emotions and the comprehension of second-order beliefs. Misailidi's (2020) study builds a bridge between the organization of shame and Theory of Mind (ToM), defined as the ability to infer the mental states of others and one's own (Premack and Woodruff, 1978), and asserts that children begin to understand the shame of others at 5 years of age. In addition, participants with higher scores on the external shame recognition task^2^ were also better at attributing second-order false beliefs. Thus, a link emerges between external shame and the way in which we hypothesize how others may see and evaluate us, or how our social self is represented in the minds of others. When we consider this, it becomes clearer that people with unusual or atypical experiences of social emotions often have difficulty developing mentalizing abilities. Heerey et al. (2003) showed a correlation between deficits in ToM and the ability to recognize self-conscious emotions in a group of boys (aged 8–15 years old) with high functioning autism. This study suggests that children who are more adept at encoding second-grade beliefs may also have a greater ability to perceive how they are viewed by others. This enhanced perspective-taking ability likely contributes to their greater social competence.

### 2.3 Comparing models

Tracy and Robins (2004) base the process model of self-conscious emotions on two assumptions; (i) Higgins' (1987) general intuition that different emotions are triggered by discrepancies in different states of the self, and (ii) the study of Carver and Scheier (1998) which holds that human behavior is a goal-directed behavior and regulated by feedback processes. It seems that a movement toward a self-regulating goal involves positive emotions and vice versa, where a movement in the opposite direction involves negative emotions. The resulting dimensional model is able to predict the elicited emotion from the feedback characteristics. Within this schema, a person exposed to an event that is not relevant for survival can focus on the self and activate self-representations. If the event is appraised as pertinent to identity-based goals and ideal-self representations, the process goes on leading to the evaluation of the identity-goal congruence. If there is a discrepancy between current states of the self and the ideal state, emotions are generated.

According to Lewis (2008), shame relates to a set of standards, rules and goals (SRGs model) that are nothing more than “inventions of the culture” transmitted to young people through teaching. This model explains self-conscious emotions as responses that arise when individuals evaluate their behavior against internalized standards, rules, or personal goals. These comparisons drive feelings such as shame, guilt, or pride, depending on whether one meets or falls short of these criteria. Evaluating how one's behavior deviates from a cultural model involves judging how well the behavior aligns with the standard. This judgment can evoke emotions, such as pride when the behavior meets or exceeds the standard and shame, embarrassment, or guilt when it falls short. Lewis (2008) also highlights the role of internal or external attribution. From the combination of these three elements – affirmation of SRGs, self-assessment intervention and attributional characteristics – the self-conscious emotions are generated. Individuals who assess that they did not adhere to a model perceive themselves as globally unable to match expectations and feel shame. This is often activated in reaction to public transgressions (Tangney and Dearing, 2002; Wolf et al., 2010). There is some overlap between the SRGs model and the process model of self-conscious emotions, although Lewis' (2008) model places a strong emphasis on the more subjective outcomes of cultural models. These two models have the merit of being able to create an interpretative grid that allows one to read the entire set of self-related emotions. However, as pointed out by Mesquita and Karasawa (2004) referring to the model of Tracy and Robins (2004), the fundamental step of appraisal is the weak link in this theory because appraisals differ across cultures and in function of different models of self. The authors explain that emotions should be interpreted as dynamic cultural processes rather than biologically based discrete entities.

Grecucci et al. (2021) conceptualize shame as a *moral self-regulatory process* (sometimes described in their earlier work as a “*moral algorithm”*) through which individuals monitor discrepancies between their behavior and internalized social or moral standards. In this perspective, shame functions as an internal signal that draws attention to aspects of the self perceived as misaligned with valued norms. Although this regulatory role can be mapped onto identifiable neurocognitive mechanisms, it should not be understood as a mechanical or deterministic procedure; rather, it reflects a dynamic, context-sensitive process integrating emotional responses, social evaluations, and self-representations.

Neuroscientific support is provided to the model by specifically analyzing how the medial prefrontal cortical area monitors and detects violations. In case of a detected violation, the network related to negative emotionality (amygdala, anterior cingulate cortex and insula) would be triggered. Finally, the dorsolateral prefrontal area would be activated to inhibit the undesired behavior (Piretti et al., 2020, 2023). This model also makes clinical predictions when the algorithm works in an abnormal way (Grecucci et al., 2021). If shame is excessively reduced, the brain fails to adjust the self to the social and moral standards (e.g., to provide a negative feedback), and the individual may display antisocial, narcissistic and criminal behaviors as a consequence (aggression, rage, selfishness, exploitation, difficulty to stick to social and moral norms, and so on). In other words, the algorithm fails to inhibit morally and socially reprehensible aspects of the self.

## 3 The relational self

### 3.1 First phases of the development of self

The models presented have the self as the main protagonist. The self is at the center of the person's cognitive evaluation and violation of the integrity of this representation can lead to a strong emotional reaction, which depends on several developmental factors (self-esteem, attachment style, ideal self, ability to mentalize). It is therefore important to clarify how the self is structured, what the developmental path is, and the environmental factors that influence this multifaceted construct (McConnell et al., 2012).

The following will outline the main stages of self-development in relation to the social other, considering four developmental periods: 0–6 months, 6–12 months, 12–18 months, and 18–24 months. Within these periods, the key and fundamental milestones that enable the child to develop a relational self will be highlighted.

#### 3.1.1 0–6 months: dyadic mirroring; emotional attunement

The self is both embodied and embedded in social interaction (e.g., Goffman, 1959; Schegloff, 2007; Tantucci, 2021). Considered under the embodied view, it is often confused with self-consciousness. The two concepts are related but not synonymous, as the self encompasses a wider range of experiences – as shame – and qualities, while self-consciousness is a more specific aspect of the self that develops over time. The self has sensory and motor foundations (Riva, 2018; Salomon, 2017). It can be argued that a bodily sense of the self develops as early as the late fetal period when the *fetus* is able to perceive the touch of her/his own limbs, thus with proprioception (Aitken and Trevarthen, 1997; Delafield-Butt and Ciaunica, 2024). Neisser (1995) speaks of ecological self to define the self as what is directly perceived with respect to the immediate physical environment. In fact, the analysis of the rooting reflex in the infant has made it possible to highlight how the infant is able to distinguish between a self-stimulation and an external stimulus (Rochat and Hespos, 1997).

In the first months of life, the self is implicitly experienced as a sense of efficacy in the actions that provide feedback to the infant (Case, 1991). When children act on objects, they receive multi-sensory feedback. Through the processing of the sensations received from sense organs and the temporal juxtaposition of physical events, children experience their own agency and begin to infer the casual relationships and rules that affect the physical world. Actions are not only oriented toward objects, but facial expressions are also included. Mirroring mechanisms play a key role in triggering imitative processes in children (e.g., Rizzolatti and Fabbri-Destro, 2010; Rochat and Zahavi, 2011). Along with other aspects of cognitive development, simple imitation evolves gradually into more complex social cognitive processes. These processes progress from the “emergent self”, which Stern (1985) defined as a stage reflecting the infant's ability to initiate, regulate, and conclude interactions with their caregiver, to the development of the “core self”. This occurs between the second and 6 months, when the infant gains an understanding of being physically and causally separate from others. Around the first year of life, the use of an anticipatory smile to elicit a positive reaction in the caregiver demonstrates an active management of this ability (e.g., Messinger and Fogel, 1998; Venezia et al., 2004).

#### 3.1.2 6–12 months: joint attention and social referencing

Many scholars have emphasized the importance of being-in-relationship with others as a foundation upon which to build the self. The primal relationship experience for the infant is the relationship with the mother. The innate intersubjectivity (Trevarthen, 1998), starting with the search for eye contact and matching emotional expressions through continuous empathic mirroring is closely related to what Fogel (1995) calls *the relational self* . The processes that organize the infant's experience of self are not only embodied, but embedded in relational activity between the child and adult. Around the 9 month of life, with the emergence of secondary intersubjectivity, “dyadic states of consciousness” are expanded to include external objects and imply new and more complex levels of social-affective relations whose outcome determines the proper organization of the self (Fonagy and Allison, 2014). The fundamental role of intersubjective communication emerges at this specific stage and allows the child to expand the dimension of the self from the sensorimotor field to the relational communicative field (Trevarthen, 1998). Through this triangular dynamic activity of observation, imitation, and joint attention (Mundy and Newell, 2007), children learn to understand not only their own thoughts and feelings, but also those of others and the relationships between objects and events. Children experience an emotional resonance with caregivers that allows them to move from a neutral affect state to one with high arousal and positive state. Intersubjective attunement involves a multimodal sensory amplification of the child state. This articulated process of mutual interpersonal synchronization and attunement (Stern, 1985; Trevarthen, 1998; Tronick, 1989) supports the child's emerging sense of self, self-consciousness, and social awareness (Feldman, 2007; Stern, 1985). The emotional dimension is not the only one that benefits from relationships. A mechanism of linguistic resonance emerges between children and caregivers—one that is dynamically and creatively co-constructed as interlocutors use verbal expressions that are formally and phonetically similar to those of a prior speaker (Tantucci and Wang, 2021, 2023).

Intersubjectivity does not play a prominent role only under its synchronism aspect. As Schore (1998) notes, misattuned face-to-face interactions that begin to occur in the second year of life inhibit positive states of intersubjectivity and are the prelude to shame. In this view, disrupted synchronicity becomes the precursor agent for a new emotion. The self, at first experienced within the boundaries of one's own corporeality, is enriched with the communicative-relational characteristics (Riva, 2018), including its failures, that will accompany infants for the rest of their life. The paradigm shift is between the intracorporeal, that is intrapersonal, and the intercorporeal (Merleau-Ponty, 1968), that is interpersonal. The abandonment of the ego-centered perspective, of the *I-positions* (Fogel et al., 2002), opens the way to the representation of the other than oneself and lays the foundation of the ToM and narrative capacity, while marking the first step toward the formation of a self in relation.

#### 3.1.3 12–18 months: recognition of self in mirror and basic sense of self-other differentiation

Beginning at 15 months, children recognize their own reflected image in the mirror (e.g., Brooks-Gunn and Lewis, 1984; Lacan, 1949). Self-recognition forms the cornerstone of self-awareness. Recognition is first based on contingent characteristics, such as the synchronicity of reflex movements, and later on recognition based on morphological clues will appear (Schaffer, 1996). Lewis (1990) suggests the presence of an *existential self* , linked to the awareness that we are beings separate from others, and a *categorical self* , linked to the awareness that we are part of the world and have specific characteristics. The development of the concept of self passes through self-consciousness that allows the individual to distinguish between what pertains to the subject and what is other than self, where the other than self is not the simple phenomenal world object but rather the complex recognition of other selves, other subjects. We are faced with a paradigm shift: what in the perinatal phase was simple self-perception is now at a different evolutionary level, inside a cognitive stage. Self-awareness allows children to perceive themselves as one of the many selves capable of weaving relationships. The development of symbolic thinking and language-based communication gives rise to what Stern (1985) called the “verbal self” enabling a richer and more articulated expression of the self. At the same time, the relational stimulus continuously feeds this dynamic of co-construction of the self. Awareness of oneself, both at the body and relational levels, and of others, allows perception of similarities and differences in others that contribute to shaping self-consciousness. This is a fundamental step that will lead to the possibility of social confrontation and the development of self-related social emotions. At this stage children start to build that image of the ideal self whose violation triggers feelings of shame, as proposed in the models seen earlier.

#### 3.1.4 18–24 months: internal working models and use of evaluative language

According to Attachment Theory (Bowlby, 1969), around the second year of life, children begin to form internal working models of the relationship with their caregiver. These attachment representations constitute an organized system of behaviors useful to satisfy biological and regulatory needs, such as the need for security, protection, stress management. Assuming that these internal working models are hierarchically structured representational systems arranged at different levels of generalizability, Thompson (2000) describes internal working models as an integrated model based on the concomitant development of implicit memory, event representation, autobiographical memory, ToM, and other functions related to social functioning. As a consequence, there seems to be a coordination in the ontogeny of all these elements that evolutionarily drives the individual toward the maturation of social skills. Internal operating models would be acquired not only through direct experiences of relating with significant others, but also from secondary representations of language-mediated experiences through parent-child dialogue (Bretherton and Munholland, 2008), an aspect that seems particularly relevant to our discussion. Representations include both cognitive and emotional components and allow the child to encode and understand situations in relationships with significant others that tend to occur with some constancy. Children can then form rules capable of driving their behavior allowing to anticipate future actions. The theory elaborated by Bowlby (1969) is directed toward the understanding of a further aspect of the self: the *social self* (Mead, 1913). The internal working models will determine the relational styles that the person brings into play in different social contexts, participating in the construction of the relational aspect of the self that could be exposed to shame. Possessing normative patterns of reference allows the child to predict behaviors that violate these patterns anticipating possible emotional responses related to self-conscious emotions. According to the Social Self Preservation Theory (Gruenewald et al., 2004), when individuals face circumstances that threaten their social value or standing, it triggers heightened emotions of diminished social worth; these situations can lead to a decrease in social self-esteem. From this perspective, the threat to the social self is an important elicitor of the experience of shame.

## 4 The cognitive self

### 4.1 From self-esteem to the ideal self

The different factors that have been reviewed until now, from self-perception to self-consciousness, and to the social self, contribute to the construction of the more general concept of self. The representation that individuals have of themselves is not a pure and self-centered view. It is rather a composition of personal and others' answers to the question: “Who am I, how do I perceive myself and how do others see me?” (Pfeifer and Peake, 2012; Tantucci, 2021; Tomasello, 2003, 2008, 2018). It is a construction. Children's vision of themselves goes through very articulated passages that always imply the presence of the gaze of others. Cooley in 1902 describes the self as a “*looking glass self* ” (for a past and contemporary review on the self, see Swann and Buhrmester, 2012). In everyone's definition of self, the gaze of the other is always present, implicitly or explicitly. Children are immersed in an environment that continuously gives them feedback on their own behavior, on their own being in relation to others, and this contributes to determining the image of themselves. If the social response is negative, the mirror will reflect a darker image of the self.

Harter (2012) emphasizes how the self-concept derives from the intricate dynamic of judgments that arise from individuals and the environment in which they live, thus implying cross-cultural differences. The outcome of this articulation is the concept of self-esteem. In early childhood, it is still too early to talk about self-esteem relative to specific skills, since these skills have not been fully acquired yet. During the preoperational stage children attribute almost exclusively positive qualities to themselves. As Harter (2006) notes, an unrealistically positive self-image may play an important developmental role in motivating the child's small achievements and providing support with respect to any failures. Environmental reinforcements play a major role at this stage. During this period, one can observe behaviors that can already predict the direction of self-esteem in the child. Shortly before the age of two, children are able to anticipate an adult's reaction by seeking positive responses to their successes and avoiding negative responses when they fail (Stipek et al., 1992). The responses that children receive from peers and adults allow them to modify their characteristics. This process allows the organization of representations of the self, leading to the full internalization of external reactions, permitting children to take the autonomous evaluation of their own performance following a type of moral algorithm (Grecucci et al., 2021).

The period of middle childhood in which the child enters primary school marks the acquisition of a series of specific skills that will lead the child toward a rapid development of self-representation. The child begins to use social comparison (Suls et al., 2002), which had already begun in early childhood, limited to caregivers, as an instrument of self-evaluation. This comparison combined with the development of representational and abstract capacity contributes to the formation of ideal models (Harter, 2003, 2012). It also improves the ability to distinguish real from ideal self-perceptions. Nevertheless, 8-year-old children, when describing themselves, rarely make reference to aspects of the ideal self, as this information is still tied to external sources. At this age, the child differentiates self-narratives based on social context by demonstrating a developed private self and public self (Uszyńska-Jarmoc, 2004). Kagan (1981) suggests that a child's developing sense of standards and self-awareness provides the foundation for understanding cultural norms. A child's impulse to imitate adults, and their distress when they fail to meet perceived standards, reflects an early internalization of the social expectations and norms transmitted culturally. Children begin to internalize their parents' values and, as described in Lewis's SRG model (2008), develop behavioral standards they feel they must follow, driven by the awareness that they may be judged according to these standards (Harter, 2012). This complex dynamic is linked to a more mature perspective taking. This is a very critical period; if the parents, for example, nurtured excessively high or unrealistic standards, children would live in continuous frustration of their own goals, which would end up structuring a negative perception of the self, remaining stranded in the dimension of imperfection (Harter, 2012). This dynamic could underlie the functional mechanism for the activation of shame.

### 4.2 The role of theory of mind in the construction of the self

According to Trevarthen (1998), individuals are constantly evaluated by attachment figures. Positive evaluations lead to feelings of pride, while negative evaluations can result in negative states, such as shame. The ability to perceive oneself as being evaluated by others is linked to the development of metacognitive skills, such as understanding what others expect of us and experiencing ourselves from their perspective. This process helps individuals build self-awareness, self-consciousness, and social awareness.

The structuring of these aspects of self and of self-conscious emotions mature in a triangular relationship together with the acquisition of the Theory of Mind (Premack and Woodruff, 1978). Through the paradigm of the False Belief Task, Wimmer and Perner (1983) showed for the first time that from the age of 4 to 6, children can understand that people may have false representations. In recent years, the age of first comprehension of others' mental states has been advanced to 3 years (Clements and Perner, 1994) and to children aged 20 months (using implicit paradigms in which the looking behavior of young participants is assessed following manipulations of the dynamic cues within the event stimuli; Surian and Franchin, 2020), 15 months (e.g., Baillargeon et al., 2010) and even 10 months (Luo, 2011). These experimental findings suggest that children begin to form expectations and make evaluations about themselves already from a very young age.

Several studies (e.g., Meins et al., 2002; Ruffman et al., 2002) support the fact that the precursors of preschoolers‘ abilities to infer others' mental states are to be sought in early childhood, particularly in the quality of social relations experienced, language acquisition, and family background. The development of ToM and language seem to proceed in parallel. Grazzani et al. (2018) showed that language ability not only explains the relationship between emotional understanding and ToM in 3- to 8-year-olds, but also explains the different levels of performance on ToM tasks. In addition, De Villiers (2000) notes how important language is, its structure, and thus how the adult's syntax, and semantic knowledge, is used by children to create representations useful for understanding false belief tasks. Two language acquisition styles can be distinguished; a referential style, related to the naming process, and an expressive style, learned through social interaction (Goldfield, 1987; Lieven et al., 1992). Both of these reflect the types of interactions and contexts that a child and caregiver develop together over time in their shared relationship (Goldfield, 1986). As a result, the use of different language styles seems to have an impact on children's understanding of mental states (Harris et al., 2005; Ruffman et al., 2002).

The use of emotional language, the ability to talk about one's moods, correlates with greater imaginative competence, which is strongly related to ToM and secure attachment (Fonagy and Target, 1997). Thus, a close link emerges between attachment patterns, individual differences in autobiographical memories and attentional capacity (e.g., Belsky et al., 1996). We stressed here the importance of ToM to highlight the connection between one's identity and what others may think of us. Shame can indeed result from an explicit negative evaluation that is directed toward us or from imagining what others might think of us, which is what we have defined as mentalization.

## 5 Linguistic self

### 5.1 The linguistic construction of the self

The second year of life marks a major milestone in the development of the self, facing more deeply its embedded aspect of social interaction. Beginning with language development, the child is able to express categorizations while also learning new ones. The self-narrative begins in early childhood from the categorical description of one's physical characteristics, abilities, preferences and possessions. The use of grammatical subjects builds the boundaries of self at a representational level. Alongside the “I” appears the “you”, a sort of specular “I” that makes it possible to escape from the gaze of one's own self and raises a further level of the scaffolding discussed in the ToM (e.g., Kuijper et al., 2021). The role of autobiographical narration by the caregiver creates the temporal structure to build a continuity of the self over time and stimulates autobiographical memory. This, in turn, allows children to create their own narrative, a social-cultural-linguistic *self-in-the-world* (Nelson and Fivush, 2004), upon which to build the organization of their own experiences. The same narrative style differently impacts on childrens' mnemonic capacity (e.g., Hedrick et al., 2009), i.e., a parent who narrates and explains the child's experiences provides her/him with an interpretive frame of her/his own reality, feeding the child's autobiographical memory. This will allow the child to narrate and understand herself/himself along a coherent time axis. Furthermore, linguistic interaction with adults fosters the development of semantic memory (Bowlby, 1969). Children are constantly immersed in a linguistic world which is geared toward labeling and definition, as we have seen earlier about the referential style language, and this is also reflected in the children's definition of self. Caregivers often use evaluative descriptors such as “you're a smart kid”, “you're really good,” or reference to rules and standards that contribute to the scripts formation (Tomkins, 1987), such as “good kids don't hit”, “always brush your teeth after eating”. Children's first descriptions of self will echo these phrases uttered by significant others.

As Klein (1975) observed, the view of the self as all-bad is often a result of the child's early experiences with his/her primary caregiver. If children perceive that their needs are not being met, or if they feel rejected or punished by the caregiver, these experiences may be internalized as a sense of worthlessness. The same dynamic has been evidenced in children who have experienced abuse or neglect. In these situations, adults always emphasize only the negative characteristics of the child, contributing to the linguistic construction of a me-self as entirely negative. The result of this construction is that children perceive themselves as bad. There is evidence of an impoverished self in children with histories of maltreatment, who also show poor autobiographical narrative skills (Toth et al., 1997) and a lower level of self-awareness, feeding a deficit in the ability to detect and express their inner states. Bretherton (1992) highlights that defensive exclusion can lead to defensive distortions in children. Maltreated children may escape into fantasy and create false selves to deal with intrusive caregiving and reject their true selves (Crittenden, 1994).

### 5.2 Storytelling and self-narration

Narrative and the child's linguistic ability development go hand in hand and are deeply linked to the construction of the self and the image-of-self. Vygotsky (1978) was among the first to grasp the importance of habitual participation in narrative practices, considering it a trigger to generate a social and psychological effect. Taking part in such practices allows one to develop self-evaluative and hetero-evaluative skills, promotes perspective taking and provides continuity in the space-time to one's self. According to Goffman (1974), those who listen to storytelling relive the story in the frame of a personal perspective. When a caregiver recounts a child's biographical episode, she/he takes possession of the child's story for a moment, reframes and contextualizes it, returning a narrative organized according to a different perspective, to the child.

Narrative plays a fundamental role in promoting the harmonious development of the child, and specifically the development of language, executive functions, and social-emotional processing (e.g., Hutton et al., 2017). The caregiver's narrative has the capacity to create representational relationships between subjects, explaining the other's point of view while providing examples of the functioning of a mature ToM. Furthermore, storytelling is a fundamental tool to improve the child's vocabulary and meta-representational skills (Fernández, 2013). The story's capacity to entertain, to frighten, to communicate emotions, is presented along with the narrative mode of another self and is enriched by its perspective.

The self, structured by the self-narration finds an echo in the fantastic imaginary narratives that are read to the child from an early age and function, to paraphrase Cooley (1902), as a mirrored narrative self. Such a mechanism is triggered when we compare an ideal model and a real situation. The evaluation of an event informs us about whether or not the two models are congruent, and the degree of deviation between these models triggers emotional responses and the resulting behavioral consequences, as previously discussed when talking about models. Whether defined as ideal self or “standards, rules, goals” (Lewis, 2008), what is triggered is a comparison between two representations. Listening to stories, tales and fables for example, contributes to the development of narrative thinking, defined as the cognitive capacity to interpret events by relating them to each other and giving them meaning (Bruner, 1987). Characters represented in children's stories allow young listeners to identify themselves through projective mechanisms and this mechanism is often exploited in clinical practice through projective techniques (Brody and Carter, 1982; McCrone et al., 1994). In addition to the moral lessons that can be drawn from the stories, it is also important to examine their structure more closely. In a fairy tale, for example, the developmental schema often provided is ideal or, at least, standardized (for example, the harmful action performed by the hero's antagonist will always be punished). The schema provides children with an interpretative framework for events and at the same time allows them to predict their development. This is similar to Bowlby's (1969) internal operating models. The predictability of this schema puts children in a positive imaginative state and the story will have an evolution orientated to reward the behaviors considered socially and morally convenient, while all the opposite behaviors will be punished, contributing to a moral construction of the self. Children's fiction tends to feature characters, situations, and emotions that resonate with young audiences. This means that children can relate to the characters and their struggles, joys, and growth, seeing parts of their own experiences reflected in the story. It becomes clear that the difference between what is perceived in reality, and the ideal model absorbed since infancy, creates a dissonance that undermines the child's self-image. This mismatch leads to shame of the individual, a sense of vulnerability, and a feeling of not living up to other's expectations, whenever a harmful situation is created.

## 6 Conclusions

Exploring the putative origins of the functioning of shame, we have discovered and analyzed the development and stages of self-formation. The self arises from a biological substrate through apperception followed by the emergence of self-awareness. However, its trajectory and the characteristics with which it manifests itself are the result of continuous environmental stimuli, accumulation of experiences and evaluative processes. Children, therefore, are influenced from the earliest years by the people who surround them and take care of them, by the environment in which they grow up and by the set of stimuli that will be provided (e.g., school, relationships with peers, relationships with adults). Among the elements that support the building of the self, we have seen how attachment patterns, self-esteem and, extending more to the social sphere, internalized normative models and ToM interplay in the building of self. We have also introduced the theme of storytelling conceived in the two meanings of reading stories and structuring the biography of the child, a topic perhaps not frequently discussed but certainly not marginal within the educational framework. Reading and biographical narration starting from the first months of a child's life represents an external stimulus that exerts a dual function – it supports and accelerates the neural circuits that will allow the acquisition of reading-writing (Horowitz-Kraus and Hutton, 2017), and it favors the structuring of the articulated construct called self. What appears quite clear therefore is the need for a stimulating context of growth in order to structure a functional and adaptive self. The absence or anomaly in the structuring factors creates a sense of self that can lead to psychological, emotional and relational difficulties.

On these foundations stand the self-conscious emotions or, as we have preferred to define in order to stress the central role of the self, self-related emotions. We explored the concept of shame, which serves as a connecting link between an individual's sense of self and their perception of others. Because shame is deeply influenced by cultural factors, it reflects this mutual relationship, highlighting the various ways in which it operates—much like a litmus test—that reveals underlying social dynamics. Attempting to give a universal definition of shame would capture only partial aspects and would be strongly dependent on the culture of reference. The definition given by Aristotle as “fear of having bad fame” (Aristotle, 2008, IV century B.C.E./2008, 1128b 10-15) probably captures the more western and general vision discussed above, ignoring all those nuances that emerge when self-conscious emotions are treated according to other cultural perspectives. Emotions can in fact be conceived of as a set of universal traits combined with properly cultural features (Mesquita and Karasawa, 2004; Wallbott and Scherer, 1986). We think it would be appropriate to analyze the basis of complex emotions contextualizing their development within the cultural frame specifically, because the development of the self is strictly dependent on the cultural context. In fact, differences in moral emotions between Western and Eastern cultures are attributed to individualistic and community ethics respectively. Western cultures reward independent self while Eastern cultures reinforce interdependence and group advantage (e.g., Bedford, 2004; Markus and Kitayama, 1991; Scherer, 1997; Wong and Tsai, 2007). As a consequence, shame has distinct characteristics in different cultures, with variations in eliciting events and behavioral outcomes (Bedford, 2004). Moreover, since emotions are recognized and defined verbally, and language plays a role in shaping self and self-related emotions (Barrett, 2016), their evaluation is cultural context dependent, with universal elements and variability in human cultures. This approach leads us to believe that a wide-angle look at this phenomenon is necessary and that future studies may benefit from such a multifactorial perspective, improving our understanding of emotional dynamics, especially when we consider self-related emotions such as shame. The key points of future research should therefore involve an interdisciplinary and multicultural approach.

The importance of exploring how shame develops in conjunction with the formation of the self and within social relationships becomes a valuable resource not only from a therapeutic perspective but also from an educational one. The gaze from others constantly impacts us, confronting us with an ideal of the self that is slowly shaped through adult models (the symbolic and real aspects of reality) and narrative models (the imaginary dimension). Being embedded within the social fabric, even through the experience of shame—a shame that is not toxic but one that is capable of morally guiding us—allows both children and adults to reshape their self-image, which becomes a tool for self-improvement.

## Footnotes

1 *Aidōs* can be understood as a kind of reverence or modesty that makes individuals sensitive to the opinions of others, restraining them from acting unjustly or shamefully. It is not merely negative shame; rather it is a social virtue that supports civil order by encouraging people to behave properly out of respect for others and the community. This feeling helps to maintain harmony and discourages harmful actions by fostering a sense of responsibility and self-control.For a better understanding of the concept of *Aidōs* in Plato's philosophy see Candiotto (2018).

2 The task consisted of ten gender-matched vignettes, each accompanied by simple line drawings, to assess children's attribution of shame from a third-person perspective. Five vignettes depicted situations likely to elicit internal shame and five depicted scenarios likely to evoke external shame. After each vignette, children reported how the protagonist would feel and justified their answer (Misailidi, 2020).
